# Mitragynine Reverses LPS-Induced Anxiety and Oxidative Stress in Rats via Modulation of Toll-Like Receptor 4 and Malondialdehyde (MDA)

**DOI:** 10.21315/tlsr2026.37.3.8

**Published:** 2026-10-15

**Authors:** Hanisah Muhammad Faiz, Suleiman Yunusa, Muhammad Hazim Abdullah, Zurina Hassan

**Affiliations:** 1 Centre for Drug Research, Universiti Sains Malaysia, 11800 USM Pulau Pinang, Malaysia; 2 Department of Pharmacology, Sa’adu Zungur University, Bauchi, PMB 65 Itas/Gadau, Bauchi State, Nigeria

**Keywords:** Mitragynine, LPS, Oxidative Stress, Anxiety, Toll-Like Receptor 4, Rat

## Abstract

Neuroinflammation marked by increased toll-like receptor 4 (TLR4) expression contributes to the pathogenesis of anxiety disorders. Mitragynine, an indole alkaloid derived from the kratom plant (*Mitragyna speciosa* Korth), shows promise as a potential pharmacotherapeutic agent for anxiety due to its anti-inflammatory activity. However, the mechanism involved in its anxiolytic effect remains unclear. This study investigated the effects of mitragynine on anxiety-like behaviours, TLR4 expression and oxidative stress in the brains of rats treated with lipopolysaccharide (LPS). Rats received a single 1 mg/kg LPS injection intraperitoneally (i.p.) to induce anxiety. Mitragynine was then administered at doses of 1 mg/kg, 5 mg/kg or 10 mg/kg body weight 1 hour post-LPS administration. The impact of mitragynine on anxiety was assessed using the elevated plus maze (EPM). TLR4 expression was measured using a Western blot, while oxidative stress markers (MDA, GSH, GPx, CAT) were measured using protein assays. Mitragynine ameliorated LPS-induced anxiety behaviours in the EPM. Mitragynine (5 mg/kg and 10 mg/kg) reversed the effect of LPS on TLR4 expression in the rats’ brains. All doses of mitragynine reduced lipid peroxidation in the frontal cortex of LPS-treated rats. As conclusion, the Mitragynine effects on LPS-induced anxiety may be associated with the reduction in TLR4 expression and MDA level.

**HIGHLIGHTS**

- Mitragynine ameliorated LPS-induced anxiety behaviours in theEPM.
- Mitragynine reduced lipid peroxidation by Malondialdehyde (MDA)in the brain of lipopolysaccharide (LPS) pre-treated rats.
- LPS-induced toll-like receptor (TLR)-4 overexpression wasreversed following mitragynine administration in rats.

## INTRODUCTION

Anxiety disorders (AD) are one of the most reported mental disorders worldwide (Khaiyom *et al*. 2019). AD includes social anxiety, selective mutism, panic disorder, generalised anxiety disorder (GAD) and agoraphobia. A national cross-sectional study reported a 29% prevalence risk of anxiety among 1,821 Malaysian university students (Mohamad *et al*. 2021). It is crucial to treat AD effectively, as it increases the risk of chronic diseases and suicide (Harro 2018). According to Khaiyom *et al*. (2019), current pharmacotherapy for AD includes selective serotonin reuptake inhibitors (SSRIs) and benzodiazepines (BZD). These classes of drugs are disadvantageous due to their side effects, such as increased suicidal ideation, emotional numbing, insomnia and gastrointestinal problems. Moreover, long-term use of BZD often leads to dependence and withdrawal.

Natural remedies are potential alternatives to SSRIs and BZDs. The use of natural products to treat anxiety has steadily grown over the past few decades (Yeung *et al*. 2018). Of interest, plants with anti-inflammatory and antioxidant properties are reported to be effective in reducing anxiety symptoms in preclinical studies (Arab *et al*. 2020; Zhang *et al*. 2018). It is postulated that neuroinflammation, initiated by microglial toll-like receptor 4 (TLR4) activation, plays a role in mediating anxiety (Zhang *et al*. 2018; Lee *et al*. 2020; Seguella *et al*. 2021). Toll-like receptor 4 detects pathogen-associated molecular patterns (PAMPs) such as lipopolysaccharide (LPS) and, upon activation, causes a signal cascade that eventually triggers the release of proinflammatory cytokines (Lee *et al*. 2020; Leitner *et al*. 2019; Zusso *et al*. 2019). Past studies show anxiety-associated increased proinflammatory markers in the hippocampus and frontal cortex following LPS administration (Luduvico *et al*. 2020).

One of the consequences of increased inflammatory markers is the overproduction of reactive oxygen species (ROS) (Beheshti *et al*. 2020; Fedoce *et al*. 2018). Elevated ROS disrupts the balance between pro-oxidants and antioxidants in cells (Fedoce *et al*. 2018; Atrooz *et al*. 2019). This disruption triggers lipid peroxidation, thereby producing a toxic substance called malondialdehyde (MDA) (Fedoce *et al*. 2018; Atrooz *et al*. 2019). Studies in human subjects show increased MDA in those diagnosed with social phobia and panic disorder (Nakao *et al*. 2021). Internal antioxidants like glutathione (GSH) and its enzyme glutathione peroxidase (GPx) eliminate free radicals to maintain redox balance (Fedoce *et al*. 2018; Atrooz *et al*. 2019). Both humans and animals exhibit anxiety-like behaviours when brain antioxidants are inhibited (Fedoce *et al*. 2018; Atrooz *et al*. 2019).

Kratom (*Mitragyna speciosa*, Korth) is a plant originating from Southeast Asia (Todd *et al*. 2020). Locally, kratom is traditionally consumed for its analgesic and opiate-like properties (Swogger & Walsh 2018). In Western countries, kratom has gained popularity and is typically consumed to self-treat opioid withdrawal symptoms, including anxiety (Johnson *et al*. 2020; Eastlack *et al*. 2020). Consumers have reported kratom as effective in reducing their anxiety symptoms, denoting its anxiolytic properties (Bath *et al*. 2020; Coe *et al*. 2019; Garcia-Romeu *et al*. 2020). This was supported by findings from preclinical studies, where administration of mitragynine, the main alkaloid content in kratom leaves, reduces anxiety behaviours in rodents and zebrafish models (Chen *et al*. 2022; Hazim *et al*. 2014; Yusoff *et al*. 2016). In terms of its anti-inflammatory and antioxidant properties, studies have shown that mitragynine lowers oxidative stress in the rat brain and inhibits the expression of inflammatory mediators both *in vivo* and *in vitro* (Chen *et al*. 2022; Utar *et al*. 2011).

On that account, the present study aims to observe the effects of mitragynine on LPS-induced anxiety-like behaviours, neuroinflammation induced by TLR4 expression and oxidative stress in the rat brain.

## METHODOLOGY

### Animals

Sprague Dawley rats (male, 6 weeks–12 weeks, 250 g–300 g) were purchased from the Animal Research and Service Centre (ARASC), Universiti Sains Malaysia (USM). Rats were acclimatised in a room with a 12-hour light/dark cycle and free access to clean water and food pellets for at least a week before behavioural testing. All experiments were performed between 9 a.m. and 4 p.m. in laboratories at the Centre for Drug Research (CDR), USM. The study protocol was reviewed and approved by the USM Institutional Animal Care and Use Committee [USM/IACUC/2020/(126)(1110)]

### Treatments

Mitragynine was extracted from the leaves of *M. speciosa* and isolated as previously described (Utar *et al*. 2011). This method yielded mitragynine with approximately 98% purity (Hassan *et al*. 2020). On the day of testing, LPS (L2630, Sigma-Aldrich) and mitragynine were dissolved in 20% v/v Tween 80 (Bio Basic). A dose of 1 mg/kg of LPS was injected intraperitoneally (i.p.) 2 hours prior to testing (Nandeesh *et al*. 2018; Norouzi *et al*. 2016). Three different doses of mitragynine (1 mg/kg, 5 mg/kg and 10 mg/kg) were injected i.p. 1 hour prior to testing. The doses were chosen based on the previous studies (Yusoff *et al*. 2016; Hassan *et al*. 2019).

### Behavioural Tests

#### Open field test

The impact of treatment on locomotor activity can be measured using an open field test (OFT) (Bednarczuk *et al*. 2025). The automated OFT box, consisting of a black floor and transparent walls (45 cm × 45 cm × 20 cm), was used in the study (IR Actimeter LE 8825, Panlab Harvard Apparatus). The box was connected to a control unit, and data were automatically recorded and analysed using Actitrack software version 2.7.13. Each rat was individually placed in the centre of the box and was allowed to explore for 10 min. After each trial, the apparatus was cleaned with 70% v/v ethanol. The parameters measured were total distance travelled, total activity and mean velocity.

#### Elevated plus maze

Anxiolytic properties of drugs can be measured using a behavioural model called the Elevated Plus Maze (EPM) (Choubey *et al*. 2019). It is made up of two closed arms and two open arms (50 cm × 10 cm) that are 50 cm above the ground. Each rat was placed at the centre of the four arms, facing an open arm, and was allowed to explore for 5 min. A camera was placed above the apparatus and video recordings were analysed using the SMART video tracking system (Panlab Harvard Apparatus). In between the testing of each animal, the apparatus was disinfected with 70% v/v ethanol. The parameters measured in the EPM were the number of open and closed arm entries and the percentage of time spent in the open and closed arms.

#### Protein extraction and quantification

At the end of the behavioural studies, the brain was harvested, and the hippocampus and frontal cortex (FC) were isolated and kept at −80°C until used. Equal volumes of the brain region were homogenised in a lysis buffer (T-PER, 1× protease inhibitor; Thermo Scientific). After 60 min of incubation at 4°C, the samples were centrifuged at 14,000 rcf for 10 min (Z216MK, Hermle). Supernatants were collected and transferred into clean tubes. Protein content in each sample was quantified using the Lowry assay method (Lowry *et al*. 1951). This assay follows a two-step reaction:

1. The protein reacts with copper in an alkaline solution.
2. The copper-bound protein reduces Folin reagent to produce a blue-coloured species with maximum absorbance at 750 nm.

The assay was carried out according to the manufacturer’s protocol (Life Science Group 2013). Briefly, samples were diluted in lysis buffer (1:30), and 5 μL of the diluted samples were pipetted into a 96-well plate in triplicate. Then, 25 μL of working reagent A (alkaline copper tartrate solution and surfactant solution, 1:20) and 200 μL of Folin reagent were added to the wells. After 15 min, absorbances were read at 750 nm (Multiskan Go, Thermo Scientific). A standard curve of Bovine Serum Albumin (BSA) (Thermo Scientific) containing 0.125–2.0 mg/mL protein was used for the calculation of protein concentration in each sample.

### Western Blotting

Following electrophoresis, proteins on the gel were blotted onto a polyvinylidene fluoride (PVDF) membrane through the wet transfer method. A gel sandwich consisting of a cassette, fibre pads, filter paper, PVDF membrane and gel was prepared and placed into a tank filled with transfer buffer (0.5 M Tris base, 3.84 M glycine, 20% v/v methanol and deionised water). Blotting was run at 90V for 45 min. After blotting, the membrane was blocked with 5% skim milk in Tris-buffered saline with Tween (TBST) for one hour and incubated overnight at 4°C with TLR4 (1:1000 dilution, D8L5W rabbit mAb, Cell Signalling, USA) and β-actin (1:1000 dilution, 20536-1-AP rabbit pAb, Proteintech). Subsequently, the membrane was washed with TBST for 5 min and incubated in a secondary antibody (1:3000 dilution, 7074 anti-rabbit IgG, Cell Signalling) for 48 h at 4°C. The membrane was then washed with TBST thrice, 5 min each time, before being incubated with streptavidin-HRP in TBST (1:3000 dilution) (Thermo Scientific) at room temperature for half an hour. Afterwards, the membrane was washed in TBST thrice, 5 min each time.

The protein bands for TLR4 and β-actin were visualised using an HRP-based colorimetric detection system (Opti-4CN, Bio-rad). The membrane was incubated in Opti-4CN substrate with gentle agitation at room temperature for half an hour. Signals were measured using a gel imaging system (ChemiDoc XRS, Bio-Rad) and quantified using ImageJ software. TLR4 protein expression was normalised with β-actin and expressed as a percentage.

#### Measurement of oxidative stress markers

Proteins from the FC were extracted as described above. Protein content in each sample was quantified using the Bradford assay method (Bradford 1976). In this method, proteins bind to Coomassie Brilliant Blue G-250, changing its colour from red to blue with maximum absorbance at 595 nm. 5 μL of samples were pipetted into a 96-well plate in duplicates. Then, 250 μL of Bradford reagent (acidified solution of Coomassie G-250) was added to the wells. After 5 min, absorbances were read at 595 nm (Multiskan Go, Thermo Scientific). A standard curve of BSA containing 25–500 μg/mL protein was used for the calculation of protein concentration in samples.

### MDA Measurement

Malonaldehyde (MDA) was measured as previously described (Kpemissi *et al*. 2019). The MPIA solution was prepared by mixing 10.3 mM 1-methyl-2-phenyl-indole (Tokyo Chemical Industry) in acetonitrile with 32 μM iron chloride (III) (NacalaiTesque) in methanol in the ratio 3:1. A reaction mixture was prepared by mixing 25 μL of the sample, 65 μL of MPIA solution and 15 μL of hydrochloric acid (37%) in a 96-well microplate. The mixture was then incubated at 45°C for 60 min. Absorbances were recorded at 586 nm. A standard curve comprising 0.3 nM–10 nM of 1,1,3,3-tetramethoxypropane (Sigma-Aldrich) was used for the quantification of MDA. MDA content was expressed as nM/mg protein.

#### GSH measurement

Reduced glutathione (GSH) was measured as previously described (Kpemissi *et al*. 2019). A reaction mixture consisting of 25 μL of samples, 100 μL of 0.2 M Tris-HCl (pH 8.9), 5 μL of 5,5′-Dithiobis(2-nitrobenzoic acid) (DTNB) (Sigma-Aldrich) in methanol was prepared and pipetted into a 96-well plate. After 5 min, absorbances were read at 412 nm. A standard curve of GSH (NacalaiTesque) with a concentration of 31.25–500 nmol/mL was used for the calculation of protein concentration in samples. GSH content was expressed as nmol/mg protein.

#### GPx measurement

Glutathione peroxidase (GPx) activity in the frontal cortex was measured according to a previously described method (Uti *et al*. 2020). A reaction mixture containing 20 μL of 0.4 M phosphate buffer (pH 7.0), 10 μL of 10 mM sodium azide, 20 μL of 10 mM GSH, 10 μL of H_2_O_2_ and 20 μL of the sample was prepared in clean tubes and incubated at room temperature for 10 min. Each tube was then added with 10% trichloroacetic acid (TCA) and centrifuged at 3,000 rpm for 15 min. The total of 40 μL of supernatant was collected from each tube and transferred into a 96-well plate. Thereafter, 50 μL of 0.4 mM tris-acetate buffer (pH 8.9) was added to each well, followed by 20 μL of DTNB. Immediately after, absorbances were measured at 412 nm against a deionised water blank. GPx activity was expressed as unit/mg protein.

#### Catalase activity measurement

Catalase activity (CAT) was measured according to the reaction between undecomposed hydrogen peroxide (H_2_O_2_) and ammonium molybdate (Hadwan & Abed 2016; Roustaei *et al*. 2022). 20 μL of H_2_O_2_ was added to 10 μL of sample in a 96-well plate and incubated at 37°C for 5 min. An equal volume of deionised water was used in place of the sample for standard. The reaction was then stopped by adding 18 μL of ammonium molybdate to the mixture. Absorbances were measured at 374 nm based on the maximum absorbance of the yellow complex produced (Olivares *et al*. 2019).

### Statistical Analysis

The Shapiro-Wilk normality test was employed to check the normality of data distribution. Data was reported as mean ± standard error of the mean (SEM). To compare the levels of anxiety, TLR4 expression, and oxidative stress markers between different treatment groups; a one-way analysis of variance (ANOVA) was performed, followed by Tukey’s multiple comparison test. A *p*-value < 0.05 was considered statistically significant. All statistical analyses were performed using GraphPad Prism version 9.3.1.

## RESULTS

### Effects of LPS and Mitragynine on Locomotor Activity in the OFT

A one-way ANOVA revealed significant decreases in total distance travelled (F_4, 29_ = 3.860, *P* = 0.0124; Fig. 1a), total activity (F_4, 27_ = 4.505, *P* = 0.0064; Fig. 1b), and mean velocity (F_4, 27_ = 5.379, *P* = 0.0026; Fig. 1c) in the LPS group compared to control, an effect which was not seen in mitragynine-treated rats. These results suggest that LPS reduced locomotor activity in rats.

### Effects of LPS and Mitragynine on Anxiety-Like Behaviours in the EPM

LPS exhibited a decreased number of open-arms entries (F_4, 25_ = 4.957, *P* = 0.0044) and time spent in open arms (F_4, 25_ = 2.887, *P* = 0.0430) of the elevated plus maze. However, the number of open arms entries and time spent in open arms was significantly increased when rats were given a high dose of mitragynine (10 mg/kg) an hour after LPS injection (Number of open arms entries, *P* = 0.0039; Time spent in open arm, *P* = 0.0261; Figs. 2a and 2b). In terms of closed-arms activities, LPS did not significantly change the number of entries (*P* = 0.6792) and time spent in the closed arms (*P* = 0.9415) compared to the control (Figs. 2c and 2d). Treatments with 5 mg/kg of mitragynine following LPS injection, however, showed a significant reduction in closed-arms entries compared to control (*P* = 0.0316). All doses of mitragynine did not produce any effects on the time spent in closed arms of the elevated plus maze (MIT 1 P = 0.4176; MIT 5 P = 0.3050; MIT 10 P = 0.5559).

### Effects of LPS and Mitragynine on TLR4 Expression in the Rats’ Hippocampus

One-way ANOVA reveals that LPS significantly increased TLR4 expression compared to the control (F_4, 20_ = 4.855, P = 0.0067). Mitragynine (5 mg/kg and 10 mg/kg) significantly decreased the TLR4 expression in LPS-treated rats (MIT 5 P = 0.0245; MIT 10 P = 0.0306; Fig. 3).

### Effects of LPS and Mitragynine on Oxidative Stress in The Frontal Cortex

LPS significantly exhibited a higher MDA level compared to the control (F_4, 25_ = 6.714, P = 0.0145), suggesting that LPS induced lipid peroxidation. Interestingly, rats treated with all doses of mitragynine an hour after LPS administration demonstrated significantly lower MDA levels compared to LPS alone (MIT 1 P = 0.0121; MIT 5 P = 0.0010; MIT 10 P = 0.0020; Fig. 4a). Therefore, mitragynine may possess a therapeutic effect against LPS-induced lipid peroxidation in the frontal cortex. Neither LPS nor mitragynine produced any significant effect on GSH and GPx in the frontal cortex. However, LPS showed slightly decreased GSH and GPx compared to control, while mitragynine ameliorated the effect of LPS on both markers in a dose-dependent manner (Figs. 4b and 4c). Moreover, neither LPS nor mitragynine significantly affected the level of CAT activity in the frontal cortex (Fig. 4d). In short, LPS may induce oxidative stress in the frontal cortex, while all doses of mitragynine significantly reversed the effect of LPS by reducing the MDA level in this brain region.

## DISCUSSION

The present study sought to determine the effects of mitragynine on LPS-induced anxiety-like behaviours using EPM. LPS administration can induce behavioural changes such as anxiety-like and depressive-like behaviours, locomotor activity, cognitive impairment, hyperalgesia and sleep disturbances (Banks *et al*. 2015; Dantzer *et al*. 2008; Goshen *et al*. 2007; Cunningham *et al*. 1992; Shih *et al*. 2011; Cohen *et al*. 2015). In this study, we found a significant reduction in locomotor activity using OFT in the LPS group. Locomotor activity, such as total distance travelled and movement velocity, provides a general index of central nervous system activity. The changes in locomotor activity alone cannot be used to infer anxiety without careful contextual analysis (Ellmers *et al*. 2024). For instance, decreased locomotion may result from sedation or motor impairment, whereas increased locomotion may reflect stimulant-induced hyperactivity rather than anxiolysis. Thus, to strengthen the interpretation of our OFT locomotor-based data, a complementary study using an anxiety-specific paradigm, EPM, was conducted.

Our study found a significant impact of LPS on the number of entries at the open arm of EPM. LPS caused a significant reduction in the number of entries in the open arms, which portrayed the possibility of the anxiogenic activity of the LPS. A similar pattern was found in the percentage of time spent in the open arms and is in accordance with de Gregório *et al*. (2022) findings. The anxiogenic potentials of LPS were also demonstrated by Lee *et al*. (2020) and Salmani *et al*. (2022). Additionally, Lee *et al*. (2020) reported no significant effect of LPS on closed-arms activity of the EPM, which is consistent with our findings. The total of 10 mg/kg mitragynine significantly increased both open-arms entry and % time spent in open arms; therefore, mitragynine may mitigate LPS-induced anxiety behaviours in the EPM. This finding further supports those of previous literature, implying the anxiolytic effect of mitragynine (Chen *et al*. 2022; Hazim *et al*. 2014; Yusoff *et al*. 2016). Humans mostly use the plant *M. speciosa*, from which mitragynine is derived, to self-treat mental illnesses and symptoms of opioid withdrawal, particularly anxiety. The users acknowledged the efficacy of *M. speciosa* in alleviating anxiety and advised others to give it a try (Bath *et al*. 2020; Coe *et al*. 2019; Garcia-Romeu *et al*. 2020). Therefore, mitragynine exhibits the potential to serve as an innovative remedy for anxiety. It is well-established that anxiety and stress have a detrimental effect on cognitive function (Berggren *et al*. 2017). Neuroinflammation, which modulates immune response, contributes to the development of anxiety disorders (Lee *et al*. 2020; Fedoce *et al*. 2018). Toll-like receptor 4 (TLR4) is expressed in the immune cells and functions to regulate inflammatory responses during infection (Ciesielska *et al*. 2021; Femenia *et al*. 2018). The binding of LPS to TLR4 activates a signalling cascade, freeing nuclear factor kappa B (NFκB) from IκBa inhibition, which leads to its nuclear translocation and subsequently the gene transcription of proinflammatory cytokines (Leitner *et al*. 2019; Vargas-Caraveo *et al*. 2017; Wang *et al*. 2019). Systemic LPS administration induces the upregulation of hippocampal TLR4 as well as its downstream inflammatory mediators such as NFκB, interleukin (IL)-1β, IL-6 and tumour necrosis factor (TNF)-α in rats (Lee *et al*. 2020; Wang *et al*. 2019; Khalili *et al*. 2018). Mitragynine, which is the main alkaloid content in kratom leaves, has been shown to be able to reduce anxiety behaviours in rodent and zebrafish models (Yusoff *et al*. 2016). In addition to that, previous studies (Chen *et al*. 2022; Utar *et al*. 2011) have shown possible anti-inflammatory and antioxidant effects of mitragynine, where it inhibits the expression of inflammatory mediators *in vitro* and *in vivo* as well as reduces oxidative stress in the rat brain (Chen *et al*. 2022; Utar *et al*. 2011). Thus, we asked if mitragynine would have an impact on TLR4 expression in rats pre-treated with LPS.

LPS, a macromolecule weighing 4.9 kDa (NCBI 2022), cannot pass through the blood-brain barrier (BBB), as the BBB functions as a barrier that protects the brain from potentially harmful substances in the blood (Peng *et al*. 2021). Only small (typically 400 Da–600 Da) lipophilic molecules can pass through the BBB via tight junctions between endothelial cells (Bellettato & Scarpa 2018; Pardridge 2012). However, LPS can increase BBB permeability through activation of cyclooxygenase (COX)-2 and, subsequently, the synthesis of prostaglandin (PG)-E_2_ (Kikuchi *et al*. 2019). This suggests the possibility of LPS passing the BBB through a lipid transport mechanism. In the present study, we found that LPS produced a significant increase in hippocampal TLR4, which is in line with many previous studies (Lee *et al*. 2020; Wang *et al*. 2019; Khalili *et al*. 2018). TLR4 detects pathogen-associated molecular patterns (PAMPs) such as LPS and, upon activation, causes a signal cascade that eventually triggers the release of proinflammatory cytokines (Lee *et al*. 2020). According to our data, TLR4 expression was considerably elevated by LPS and decreased by mitragynine (5 mg/kg and 10 mg/kg, *p* < 0.05). Therefore, an increase in TLR4 signalling may cause tissue damage and pathological inflammation, which in turn may aid in the aetiology of several inflammatory disorders. Mitragynine, on the other hand, has anti-inflammatory properties and can counteract the effects. Another factor that mediates inflammation is the overproduction of reactive oxygen species (ROS) (Atrooz *et al. 2*019; Chen *et al*. 2022). In a study by Matsunaga *et al*. (2017), mitragynine triggered the production of ROS as seen by elevated hydrogen peroxide levels and cell apoptosis. Therefore, high doses of mitragynine increase ROS concentration, thus activating NFκB and eventually elevating proinflammatory markers in the system (Atrooz *et al*. 2019; Chen *et al*. 2022). Few studies exist regarding the anti-inflammatory effect of mitragynine (Chen *et al*. 2022). These studies reported a decrease in proinflammatory cytokines (IL-6, IL-1β and TNF-α) following chronic mitragynine treatment. However, the present study only focuses on acute mitragynine injection in LPS-induced anxiety. This suggests that mitragynine may produce different effects when given chronically versus in a single administration. In conjunction with neuroinflammation, oxidative stress has also been implicated in anxiety disorders (Fedoce *et al*. 2018; Nakao *et al*. 2021). Oxidative stress is a condition that occurs due to intracellular imbalance between free radicals, which includes, but is not limited to, reactive oxygen species and antioxidants (Fedoce *et al*. 2018; Atrooz *et al*. 2019). Free radicals are unstable and highly reactive, tending to oxidise lipids in cell membranes, activating a chain of reactions called ‘lipid peroxidation’, resulting in the production of a toxic substance called ‘malondialdehyde’ (MDA) (Fedoce *et al*. 2018; Atrooz *et al*. 2019). Antioxidants such as reduced glutathione (GSH), glutathione peroxidase (GPx) and catalase (CAT) are endogenous molecules that scavenge free radicals to maintain redox homeostasis (Fedoce *et al*. 2018; Atrooz *et al*. 2019), thus acting as antioxidant markers. Studies have indicated that frontal cortex (FC) is one of the affected regions due to the oxidative stress action (Chen *et al*. 2022; Brown *et al*. 2005). Stressful stimuli can affect FC by altering the prefrontal dendritic structure and neuronal connectivity (Chen *et al*. 2022). We queried if mitragynine administration can modulate the level of these antioxidant markers in the rats’ FC. Our data shows that LPS increased MDA level in the FC, an effect which was wholly counteracted in the presence of mitragynine at all the doses tested (1 mg/kg, 5 mg/kg and 10 mg/kg).

This finding corroborates with the results of other scholars who reported elevated MDA levels in the rats’ FC and hippocampus following LPS administration (Arab *et al*. 2020; Beheshti *et al*. 2020; Salmani *et al*. 2022; Alshehri & Imam 2021). This elevated level of MDA in both the FC and hippocampus following LPS administration, reported by other scholars, may suggest similar neurobiology between the two brain structures (FC and hippocampus); however, in this study, the use of the hippocampus to measure the expression level of TLR4, while the use of FC to measure oxidative stress markers, is considered a limitation.

Here, we reported no alteration in the level of other oxidative stress biomarkers such as GSH, GPx and CAT in both LPS- and LPS+MIT-exposed rats. This may be due to the fact that the mechanism of LPS action across the BBB requires a longer time to induce noticeable changes in oxidative stress markers in the frontal cortex.

Our data concord with the findings of Chen *et al*. (2022), which support the idea of mitragynine’s therapeutic effect against LPS-induced lipid peroxidation. Lipid peroxidation is harmful, as it damages cell membranes and stimulates the release of pro-inflammatory markers, which causes neuronal death and behavioural deficits (Nakao *et al*. 2021; Cortez *et al*. 2021). In humans, lipid peroxidation is associated with anxiety disorders (Atrooz *et al*. 2019; Nakao *et al*. 2021). As a way to overcome damage to cell membranes caused by lipid peroxidation, the brain tries to compensate by increasing GSH in the FC (Cortez *et al*. 2021). GSH maintains redox homeostasis by reducing hydrogen peroxide into water and GSSG (oxidised form of GSH), and the process is catalysed by GPx (Cortez *et al*. 2021; Forman *et al*. 2009). Although in this study, the effect of mitragynine on antioxidant agents was not significant, we detected a trend of increasing patterns of GSH and GPx in the FC. Therefore, mitragynine may facilitate the increase in GSH and GPx activity to compensate for cell membrane damage and inflammation led on by LPS exposure.

Altogether, the study found that mitragynine attenuated LPS-induced anxiety-like behaviours in rats. Mitragynine also mitigated an increased hippocampal TLR4 expression and reversed LPS-induced oxidative stress via MDA, suggesting its antioxidant properties. The findings suggest that mitragynine may alleviate anxiety-like behaviours by modulating neuroinflammatory and oxidative stress pathways. Conclusions made in this study based on the suggested pathways are inferential rather than definitive.

## Figures and Tables

**FIGURE 1:**
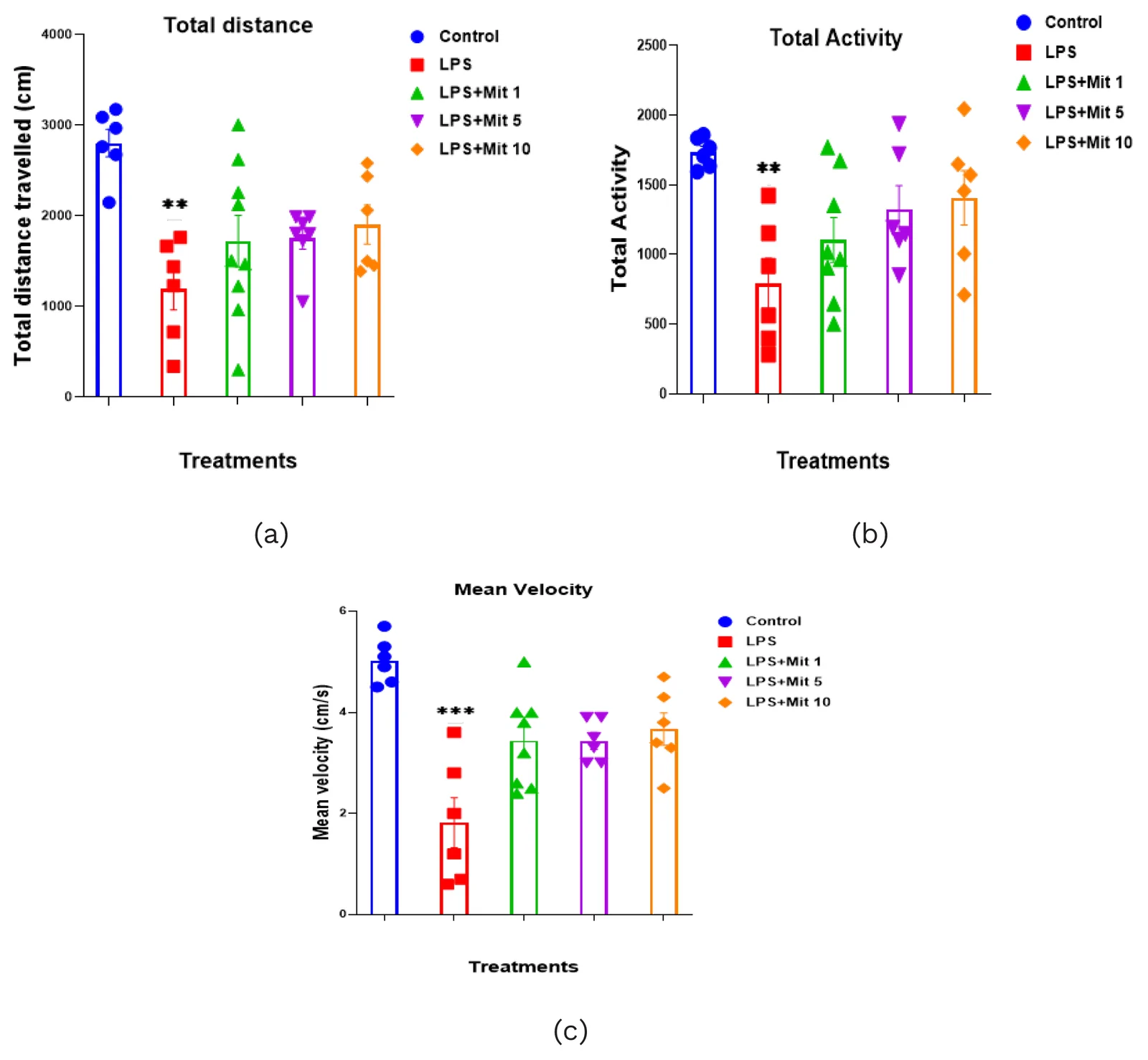
Effects of LPS and mitragynine (1, 5 and 10 mg/kg) on (a) total distance travelled, (b) total activity, and (c) mean velocity in the open field. Each bar represents mean ± SEM and was analysed using one-way ANOVA followed by Tukey’s multiple comparison test (*n* = 6 or 8 replicates/group). **p < 0.01 and ***p < 0.001 compared to control. LPS = lipopolysaccharide; Mit = mitragynine.

**FIGURE 2:**
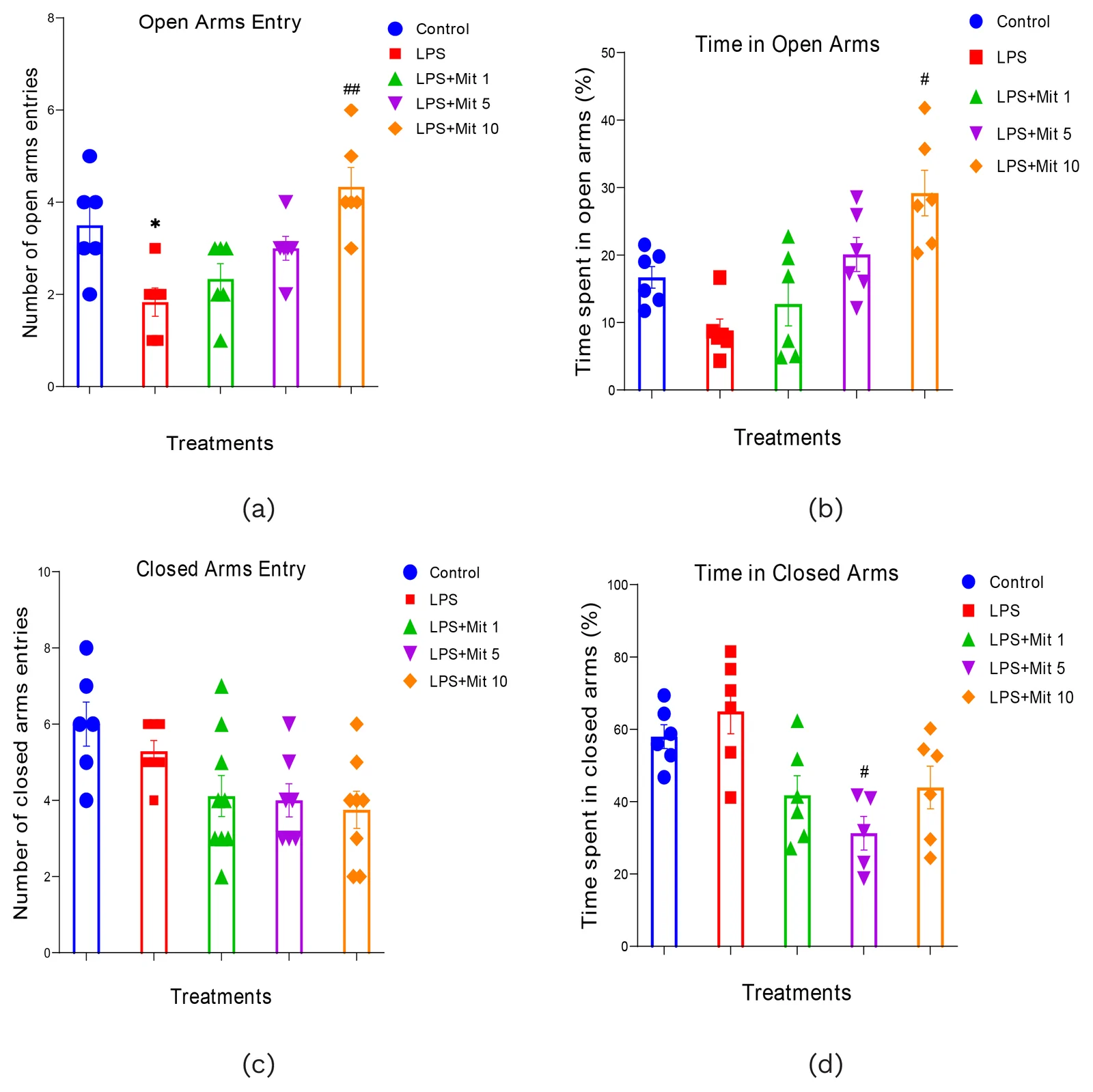
Effects of LPS and mitragynine (1, 5 and 10 mg/kg) on (a) number of open arms entries, (b) number of closed arms entries, (c) time spent in open arms, and (d) time spent in the closed arms of the elevated plus maze. Each bar represents mean ± SEM and analysed using one-way ANOVA followed by the Tukey’s multiple comparison test (*n* = 6 replicates/group). *p < 0.05 compared to control group. and #*p* < 0.05, ##*p* < 0.001 compared to LPS group. LPS = Lipopolysaccharide, Mit = mitragynine.

**FIGURE 3:**
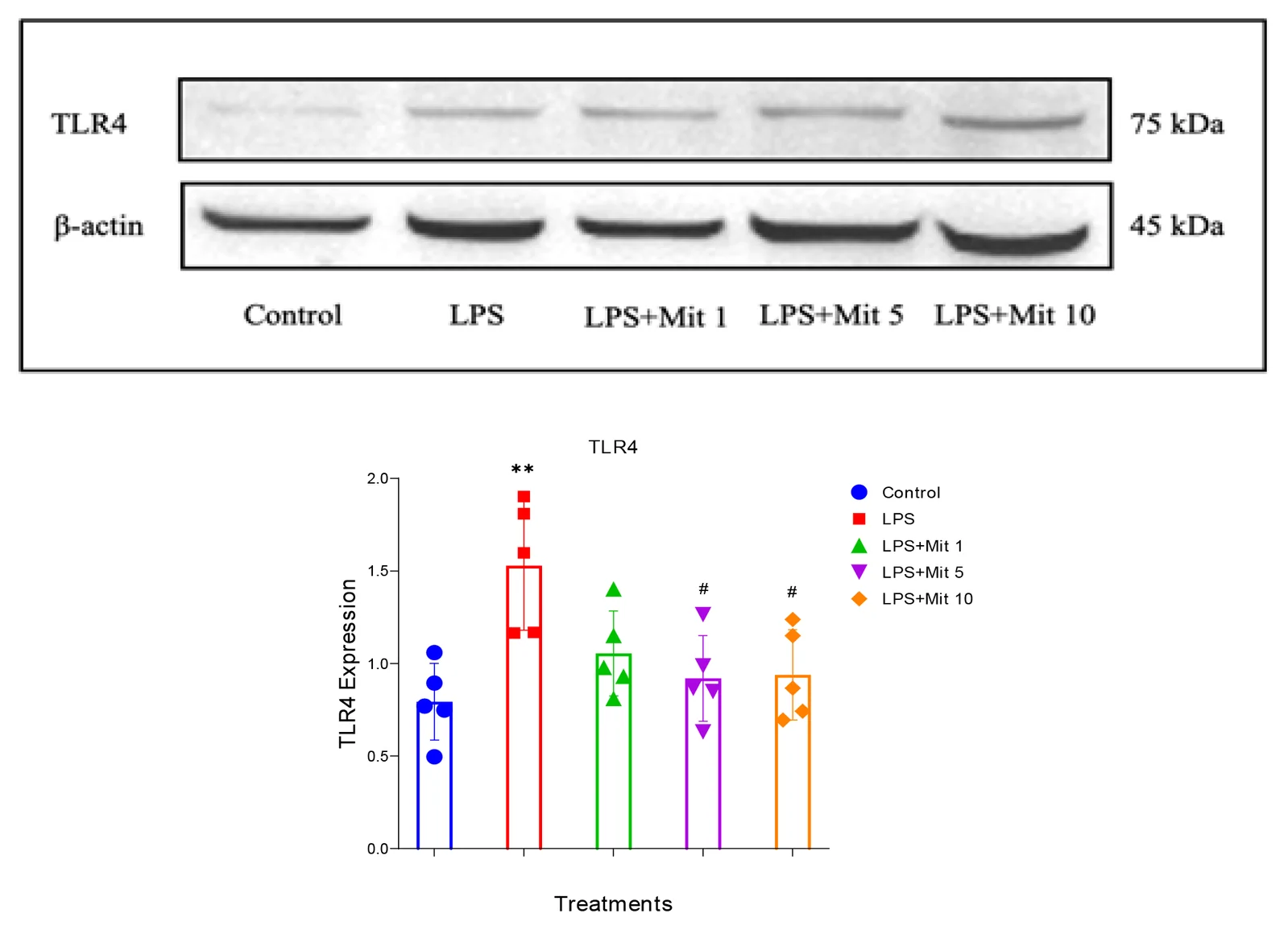
Effects of LPS and mitragynine (1, 5 and 10 mg/kg) on TLR4 expression in the hippocampus. Each bar represents mean ± SEM and analysed using one-way ANOVA followed by the Tukey’s multiple comparison test (*n* = 5 replicates/group). **p < 0.01 compared to control, and #*p* < 0.05 compared to LPS group. Values in each group were calculated in relation to the control group and beta actin served as a loading control. LPS = Lipopolysaccharide, TLR4 = Toll-like receptor 4, Mit = mitragynine.

**FIGURE 4:**
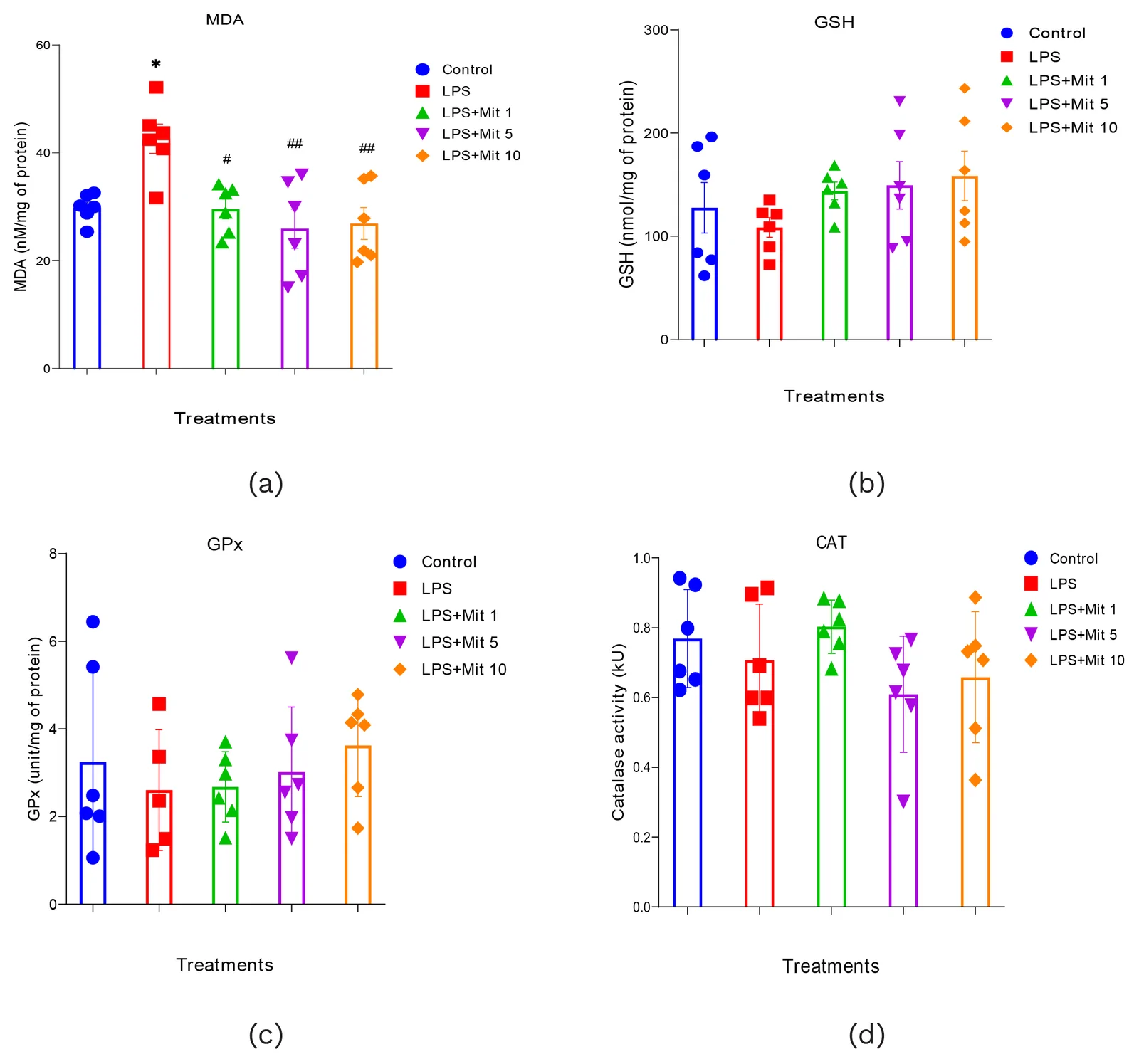
Effects of LPS and mitragynine (1, 5 and 10 mg/kg) on (a) MDA, (b) GSH, (c) GPx, and (d) CAT in frontal cortex. Each bar represents mean ± SEM and analysed using one-way ANOVA followed by the Tukey’s multiple comparison test (*n* = 6 replicates/group). *p < 0.05 compared to control, and #*p* < 0.05, ##*p* < 0.001 compared to LPS group. LPS = Lipopolysaccharide, MDA = Malondialdehyde, GSH = Glutathione, GPx = Peroxidase, CAT = Catalase, Mit = mitragynine.

## Data Availability

The datasets produced and/or analysed in this study can be obtained from the corresponding author upon reasonable request.
